# Propoxyphene Mediates Oxyhemoglobin-Induced Injury in Rat Cortical Neurons Through Up-Regulation of Active-β-Catenin

**DOI:** 10.3389/fphar.2019.01616

**Published:** 2020-01-30

**Authors:** Yuqian Li, Jiancai Wang, Zhihong Li, Hongyu Cheng, Zhuo Zhang, Tao Luo, Xingye Zhang, Guodong Gao, Huashan Lu, Lihong Li

**Affiliations:** ^1^ Department of Neurosurgery, Tangdu Hospital, Fourth Military Medical University, Xi’an, China; ^2^ Department of Ultrasound Diagnosis, Tangdu Hospital, Fourth Military Medical University, Xi’an, China; ^3^ Department of Neurology, Tangdu Hospital, Fourth Military Medical University, Xi’an, China; ^4^ Department of Emergency, 96605 Military Hospital, Tonghua, China; ^5^ Department of Emergency, Tangdu Hospital, Fourth Military Medical University, Xi’an, China

**Keywords:** Propoxyphene, active-β-catenin, apoptosis, traumatic subarachnoid hemorrhage, mitochondrial

## Abstract

Wnt/β-catenin signaling is involved in various biological processes, including the development of the central nervous system. The dysfunction of mitochondria has been shown to participate in the progress of subarachnoid hemorrhage (SAH). Traumatic subarachnoid hemorrhage (tSAH) is a serious complication in acute craniocerebral trauma. Opioids can activate the canonical Wnt/β-catenin signaling pathway. c-Myc, a downstream protein of Wnt/β-catenin signaling, contributes to the fusion of mitochondria. Here, we investigated the protective roles of Propoxyphene (Pro) against Oxyhemoglobin (OxyHb)-induced primary cultured neuron apoptosis. The data indicated that Pro rescued active-β-catenin from OxyHb-induced decline. Furthermore, Pro attenuated OxyHb-induced apoptosis and fission of mitochondria in primary cortical neurons. However, the protective effects were abrogated under active-β-catenin-deficient conditions. Together, the data presented here showed that Pro, a weak opioid analgesic drug, attenuates OxyHb-induced mitochondria-dependent apoptosis in an active-β-catenin-c-Myc-dependent manner.

## Introduction

Subarachnoid hemorrhage (SAH), which accounts for 5% of all stroke cases, is a severe neurological disorder with extremely high rates of mortality and morbidity (Boettinger et al., 2017; Ye et al., 2018). The causes of SAH are rupture of intracranial aneurysm and severe craniocerebral injury (Anzabi et al., 2018). Traumatic subarachnoid hemorrhage (tSAH), a major complication of acute craniocerebral trauma, may induce early brain injury (EBI) and elevation of intracranial pressure (Dalle et al., 2018). The general assumption is that EBI induced by a decrease in cerebral blood flow is the primary cause of poor prognosis and plays an important role in the overall outcome (Feng et al., 2016). Neuronal apoptosis, which induces disruption of the blood-brain barrier, oxidative stress, brain edema, and elevated intracranial pressure, is a common result of general EBI (Ye et al., 2018). However, the exact molecular mechanisms underlying neuronal apoptosis have not been fully studied. As a major component of blood, oxyhemoglobin (OxyHb) has been shown to be a major cause of cerebral vasospasm and decrease in cerebral blood flow (Li et al., 2016). Therefore, OxyHb has been used to mimic the pathology of SAH in primary rat cortical neurons.

Wnt/β-catenin signaling is involved in various biological processes, including proliferation and apoptosis (Wang et al., 2018). The canonical Wnt/β-catenin signaling pathway has been reported to play an important role in the development of the central nervous system (Corachan et al., 2018). With the binding of Wnt ligands to Frizzled and LRP5/6 receptors, the destruction complex assembled by Axin, APC, and GSK3β is disrupted, resulting in β-catenin protein stabilization in the cytoplasm fraction and then translocation to the nucleus fraction, where β-catenin protein binds to TCF/LEF transcription factor and drives downstream gene expression (Fan et al., 2014).

Propoxyphene (Pro) is an opioid analgesic drug intended for the treatment of mild pain (Mattoo et al., 2013). Because it is very weak in comparison to commonly abused opioids such as morphine and fentanyl, propoxyphene does not have a substantial mental craving effect. The canonical Wnt/β-catenin signaling pathway can be activated by various opioids (Wang J. et al., 2017). Furthermore, the opioids show many beneficial roles against damage in the central nervous system (Elyasi et al., 2014).

Mitochondria are vital organelles in eukaryotes and supply energy for all cellular activities (Park et al., 2018). Mitochondrial dysfunction has been shown not only in the progress of SAH models but also in SAH patients (Cai et al., 2015). Mitochondrial fission produces dysfunctional organelles, and fusion can maintain the integrity of mitochondria. Mitochondrial disruption and fission have been shown to occur after SAH (Wu et al., 2017). Furthermore, Wnt/β-catenin signaling is responsible for an increase in membrane fusion (Graves et al., 2012).

Opioids can activate the canonical Wnt/β-catenin signaling pathway (Dunbar et al., 2006). c-Myc, a down-stream protein of Wnt/β-catenin signaling, contributes to the fusion of mitochondria (Graves et al., 2012). Previous studies have shown that excessive mitochondrial fission is involved in the EBI stage following SAH (Wu et al., 2017). This study, therefore, aims to investigate the protective role of propoxyphene against OxyHb-induced cell apoptosis in primary cortical neurons.

## Materials and Methods

### Primary Cell Culture

Pregnant SD rats were purchased from the animal center of the Fourth Military Medical University. Primary rat cortical neurons were obtained from the embryos of pregnant SD rats. The brains of SD rat embryos were removed with appropriate instruments. The cortical tissues were carefully dissected and placed in Hank’s balanced salt solution. The tissues were then digested in 0.125% trypsin for 15 min at 37℃ and balanced with Dulbecco’s Modified Eagle’s Medium (DMEM; Gibco, Grand Island, NY, USA). The digested tissues were filtered through a sterile mesh filter and centrifuged at 1000 rpm for 5 min. The cells were cultured with neurobasal medium (Gibco, Grand Island, NY, USA). The primary cortical neurons were cultured to day 7 for different treatments. To establish the *in vitro* model of SAH, the cortical neurons were exposed to 10 μM OxyHb (purity≥90%, Ruibio, 07109) based on a previous study (Li et al., 2016).

### Reagents

DMEM and fetal bovine serum (FBS) were purchased from Gibco (Gaithersburg, MD). Propoxyphene (1 mg/ml, ampule of 1 ml) and MTT (purity≥98%) were obtained from Sigma-Aldrich (St. Louis, MO). Oxyhemoglobin (purity≥90%, Ruibio, 07109) was obtained from Bomei Biotechnology Co., Ltd. (Hefei, China). MitoTracker Red was purchased from Life Technologies (Massachusetts, USA). Antibodies to active-β-catenin, actin, and cleaved caspase-8 were purchased from Cell Signaling (Beverly, MA, USA). Antibodies to β-catenin, Bcl_2_, Bax, and c-Myc were purchased from Abcam (Cambridge, UK). Anti-mouse-HRP IgG and anti-rabbit -HRP IgG were obtained from Biosynthesis Biotechnology (Beijing, China).

### Transfection

Primary rat cortical neurons were seeded in 6-well plates. The cells were then transfected with β-catenin siRNA or negative control siRNA (GenePharma, Shanghai, China) using Lipofectamine 2000 for 48 h (Liu et al., 2017). The final concentration of siRNA was 50 nmol/L, and the β-catenin siRNA sequences are shown in **Table 1**.

**Table 1:** The siRNA sequences targeting β-catenin.

Negative Control siRNA	Sense 5’-UUCUCCGAACGUGUCACGUTT -3’
	Antisense 5’-ACGUGACACGUUCGGAGAATT-3’
β-catenin siRNA1	Sense 5’-GCUGUUCUAUUCCGAAUGUTT -3’
	Antisense 5’-ACAUUCGGAAUAGAACAGCTT -3’
β-catenin siRNA2	Sense 5’-GCCUUAGUAAACAUAAUGATT -3’
	Antisense 5’-UCAUUAUGUUUACUAAGGCTT -3’

### Western Blot Analysis

Primary rat cortical neurons were suspended in lysis buffer after treatment. Equivalent amounts of protein were then loaded and separated by sodium dodecyl sulfate-polyacrylamide gel electrophoresis (SDS-PAGE) and transferred to immobilon nitrocellulose (NC) membranes (Millipore, Boston, USA). The NC membranes were blocked with 5% BSA in TBST at room temperature for 2 h and incubated with primary antibodies overnight at 4°C. The membranes were then incubated with horseradish peroxidase-conjugated secondary antibodies for 2 h at room temperature. The protein bands were detected using a Bio-Rad imaging system (Bio-Rad, Hercules, USA).

### TUNEL Staining

Primary neuron apoptosis was analyzed using terminal deoxynucleotidyl transferase dUTP nick-end labeling (TUNEL) assay. Briefly, after being fixed in 4% formaldehyde solution in PBS for 20 min and permeabilized with 0.2% Triton X-100 for 10 min, the cortical neurons were incubated with TUNEL reaction mixture at 37°C for 1 h. After being washed three times with PBS, the slides were incubated with DAPI for 5 min in the dark. Finally, the TUNEL-positive cells were examined with a fluorescence microscope (C2 Si; Nikon, Japan).

### Immunofluorescence

Cortical neurons were grown on the slides and fixed in 4% formaldehyde solution in PBS for 10 min. After permeabilization with 0.2% Triton X-100 for 30 min and blocking with 5% BSA for 2 h, the primary cortical neurons were incubated with primary antibodies at 4°C overnight. The cells were then washed with PBS three times and labeled with secondary antibodies. The nuclei were labeled with DAPI. Fluorescence images were obtained with a fluorescence microscope (C2 Si; Nikon, Japan).

### Mitochondrial Staining

The morphology of mitochondria was detected by MitoTracker Red according to the manufacturer’s instructions. Briefly, cortical neurons were grown on slides and stained for 30 min with 10 nM MitoTracker Red at room temperature. Mitochondria were imaged under a fluorescence microscope (C2 Si; Nikon, Japan).

### Measurement of Cell Viability

Cell viability was determined by MTT assay. Primary cortical neurons were cultured in 9-well plates. After the designed treatment, the medium was replaced with MTT solution for 2-4 h at 37°C. The MTT solution was then discarded, and 150 μl DMSO was added. Absorbance was read at 490 nm using a microplate reader (Bio-Rad, Hercules, CA, USA). Cell viability was calculated using the following formula: cell viability (%) = optical density in test well per experiment/optical density in control well per experiment×100%.

### Statistical Analysis

All results are expressed as mean ± SEM. Prism 5 (GraphPad Software, San Diego, CA, USA) was used to analyze the data in our study. Statistical analysis was conducted using one-way analysis of variance (ANOVA) followed by Bonferroni test for multiple groups. The value of P < 0.05 was considered to be statistically significant.

## Results

### tSAH-Induced Rat Cortical Neuron Apoptosis

**Figure 1:**
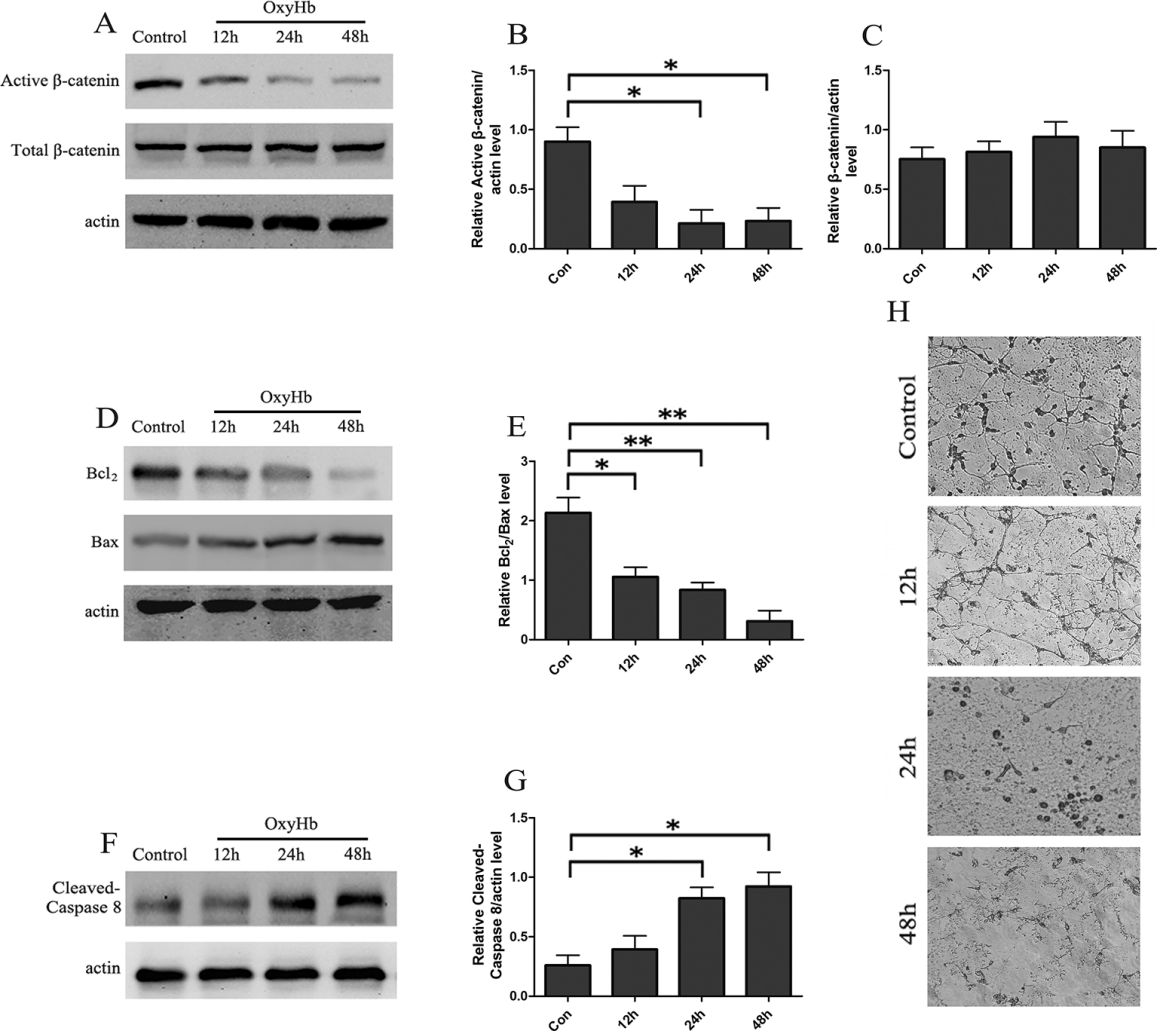
tSAH induced apoptosis and inactivation of Wnt signaling in primary cultured neurons. **(A)** Western blot analysis of active-β-catenin and β-catenin in primary cultured neurons at 12, 24, and 48 h after OxyHb exposure. **(B**, **C)** Relative amounts of active-β-catenin/actin and β-catenin/actin (mean ± SD; n = 3; *p < 0.05). **(D)** Western blot analysis of the Bcl2/Bax level in primary cultured neurons at 12, 24, and 48 h after OxyHb exposure. **(E)** Relative Bcl2/Bax values (mean ± SD; n = 3; *p < 0.05; **p < 0.01). **(F)** Western blot analysis of cleaved caspase-8/actin level in primary cultured neurons treated as described in A. **(G)** Expression of cleaved caspase-8 (mean ± SD; n = 3; *p < 0.05). **(H)** Morphological changes in primary cultured neurons (scale bar, 50 μm).

A cell tSAH model was established by adding OxyHb to the rat cortical neurons. The Western blots indicated that the levels of active-β-catenin were gradually decreased in the rat cortical neurons at 12, 24, and 48 h following OxyHb treatment and that the relative protein level of total β-catenin was stable (**Figures 1A–C**). Besides, the results showed that, compared to the control group, the ratio of Bcl2/Bax was significantly decreased in rat cortical neurons 12, 24, and 48 h after OxyHb treatment (**Figures 1D**, **E**). Furthermore, the level of cleaved caspase-8 increased markedly and remained stable during the OxyHb treatment compared with that of the control group (**Figures 1F**, **G**). The study of morphological changes in rat cortical neurons indicated that neurons showed a loss of condensation of the soma, a shrinkage of the neuron and neuronal arborization after 12, 24, and 48 h of OxyHb treatment (**Figure 1H**).

### Pro Rescued Active-β-Catenin From the OxyHB-Induced Decline

**Figure 2:**
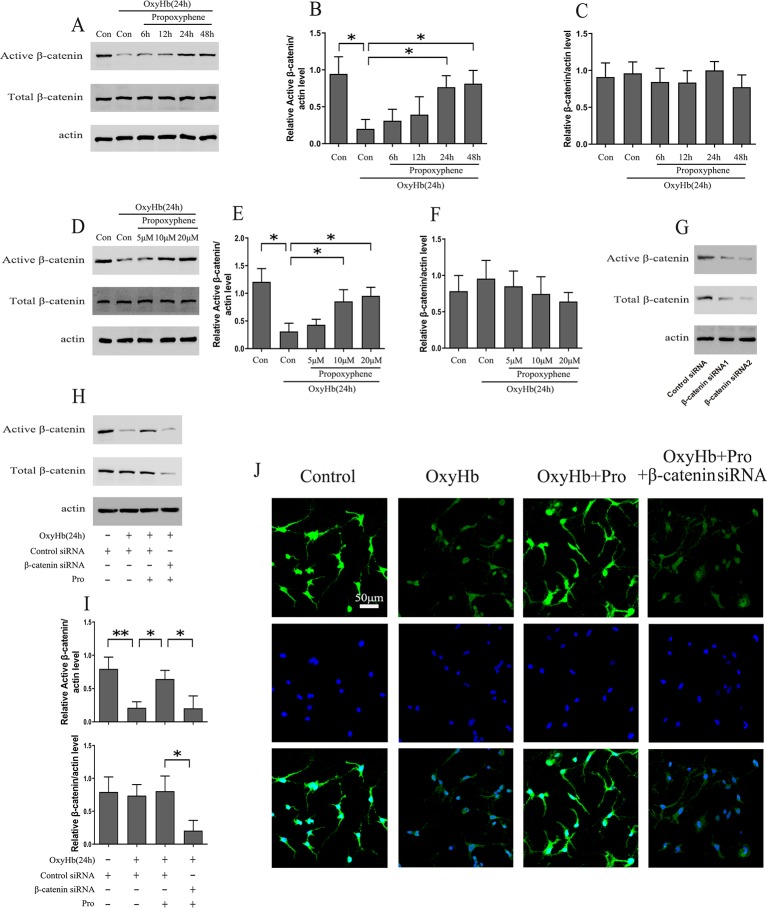
Pro rescued active-β-catenin from OxyHb-induced decline. **(A)** Primary cultured neurons exposed to OxyHb were pretreated with 10 μM Pro for 6, 12, 24, or 48 h, and Western blots were conducted. **(B**, **C)** Relative amounts of active-β-catenin/actin and β-catenin/actin (mean ± SD; n = 3; *p < 0.05). **(D)** Western blot analysis of active-β-catenin and β-catenin level in primary cultured neurons pretreated with 5, 10, or 20 μM Pro for 24 h before adding OxyHb. **(E**, **F)** Relative amounts of active-β-catenin/actin and β-catenin/actin (mean ± SD; n = 3; *p < 0.05). **(G)** Western blot analysis of active-β-catenin and β-catenin level in primary cultured neurons transfected with β-catenin siRNA. **(H)** Cells transfected with β-catenin siRNA were treated with Pro before adding OxyHb, and Western blots were conducted. **(I)** Relative amounts of active-β-catenin/actin and β-catenin/actin (mean ± SD; n = 3; *p < 0.05; **p < 0.01). **(J)** Immunofluorescence analysis of active-β-catenin in primary cultured cells treated as described in H (scale bar, 50 μm).

Rat cortical neurons exposed to OxyHb were pretreated with 10 μM Pro for 6, 12, 24, and 48 h. The results indicated that the level of active-β-catenin reached a maximum concentration after 24 h of Pro treatment before adding OxyHb compared with cells treated with OxyHb alone and that the level of total β-catenin was stable (**Figures 2A–C**). Further study that took into consideration the concentration of Pro showed that the level of active-β-catenin in cells pretreated with 10 or 20 μM Pro for 24 h before adding OxyHb was approximately 2.5 times higher than that of cells treated with OxyHb alone (**Figures 2D–F**). Primary cortical neurons were transfected with β-catenin siRNA1 or β-catenin siRNA2, and the results showed that the β-catenin siRNA2 worked effectively (**Figure 2G**). The β-catenin siRNA2 was then used in the subsequent experiments. The Western blots showed that Pro could reverse the down-regulation of active-β-catenin induced by adding OxyHb and that silencing β-catenin largely abrogated the positive effect of Pro in primary cortical neurons (**Figures 2H, I**). Furthermore, the immunofluorescence results also indicated that Pro reversed the down-regulation of active-β-catenin induced by adding OxyHb and that silencing β-catenin abrogated the positive effect of Pro in primary cortical neurons (**Figure 2J**).

### Pro Attenuated OxyHB-Induced Apoptosis in Primary Cortical Neurons *via* Wnt Signaling

**Figure 3:**
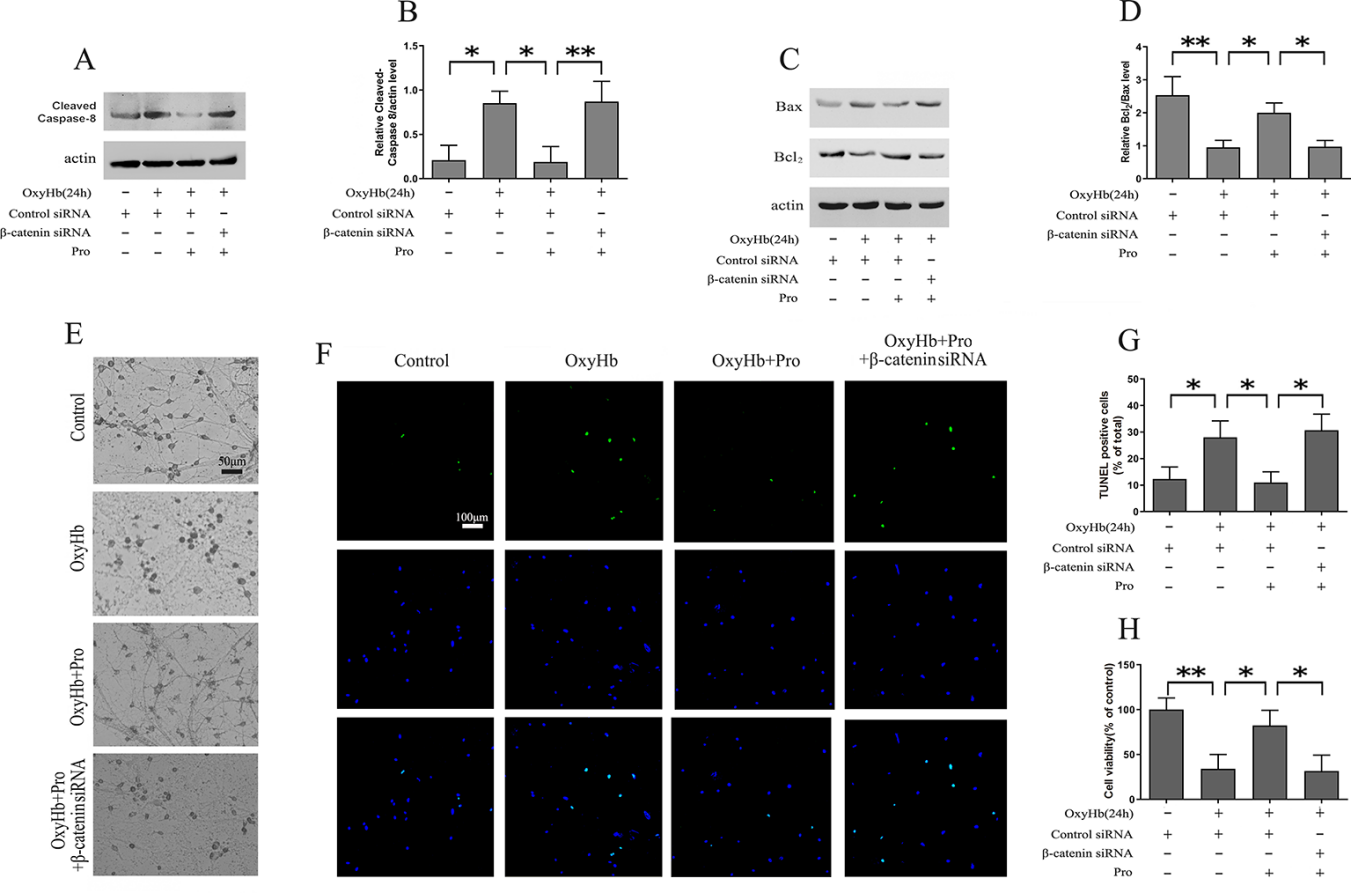
Modulation of primary cortical neuron apoptosis by Pro *via* Wnt signaling. **(A)** Western blot analysis of the cleaved caspase-8/actin level in primary cultured neurons treated as described in **Figure 2H**. **(B)** Expression of cleaved caspase-8 (mean ± SD; n = 3; *p < 0.05; **p < 0.01). **(C)** Western blot analysis of Bcl2/Bax level in primary cultured neurons treated as described in **Figure 2H**. **(D)** Relative optical density of Bcl2 normalized to Bax (mean ± SD; n = 3; *p < 0.05; **p < 0.01). **(E)** Morphological changes in primary cultured neurons treated as described in **Figure 2H** (scale bar, 50 μm). **(F)** Primary cultured neurons stained for TUNEL (scale bar, 100 μm). **(G)** Ratios of TUNEL-positive/Total primary cultured cells (mean ± SD; n = 3; *p < 0.05). **(H)** Results of MTT assay in primary cultured cells treated as described in **Figure 2H** (mean ± SD; n = 3; *p < 0.05; **p < 0.01).

Western blots indicated that OxyHb treatment markedly increased the expression of cleaved caspase-8. The pretreatment of Pro ameliorated the activation of cleaved caspase-8. Conversely, knockdown of β-catenin largely abrogated the protective effect of Pro against OxyHb-induced activation of cleaved caspase-8 (**Figures 3A, B**). The same conclusion was obtained when we analyzed the Bcl2/Bax ratio in Western blots (**Figures 3C, D**). The study of morphological changes in rat cortical neurons indicated that the loss of condensation of the soma, shrinkage of the neuron, and neuronal arborization induced by OxyHb treatment could be attenuated by Pro treatment and that silencing β-catenin largely abrogated the positive effect of Pro (**Figure 3E**). The TUNEL staining indicated that OxyHb treatment induced the up-regulation of TUNEL-positive cells and that Pro pretreatment before adding OxyHb induced a significant decrease of TUNEL-positive cells compared with the OxyHb treatment group. However, silencing β-catenin abolished the protective effects of Pro, as indicated by the change in TUNEL-positive cells (**Figures 3F, G**). The same conclusion was obtained in the MTT assay (**Figure 3H**).

### OxyHB-Induced Mitochondrial Fission Could be Prevented by Pro *via* Wnt Signaling

**Figure 4:**
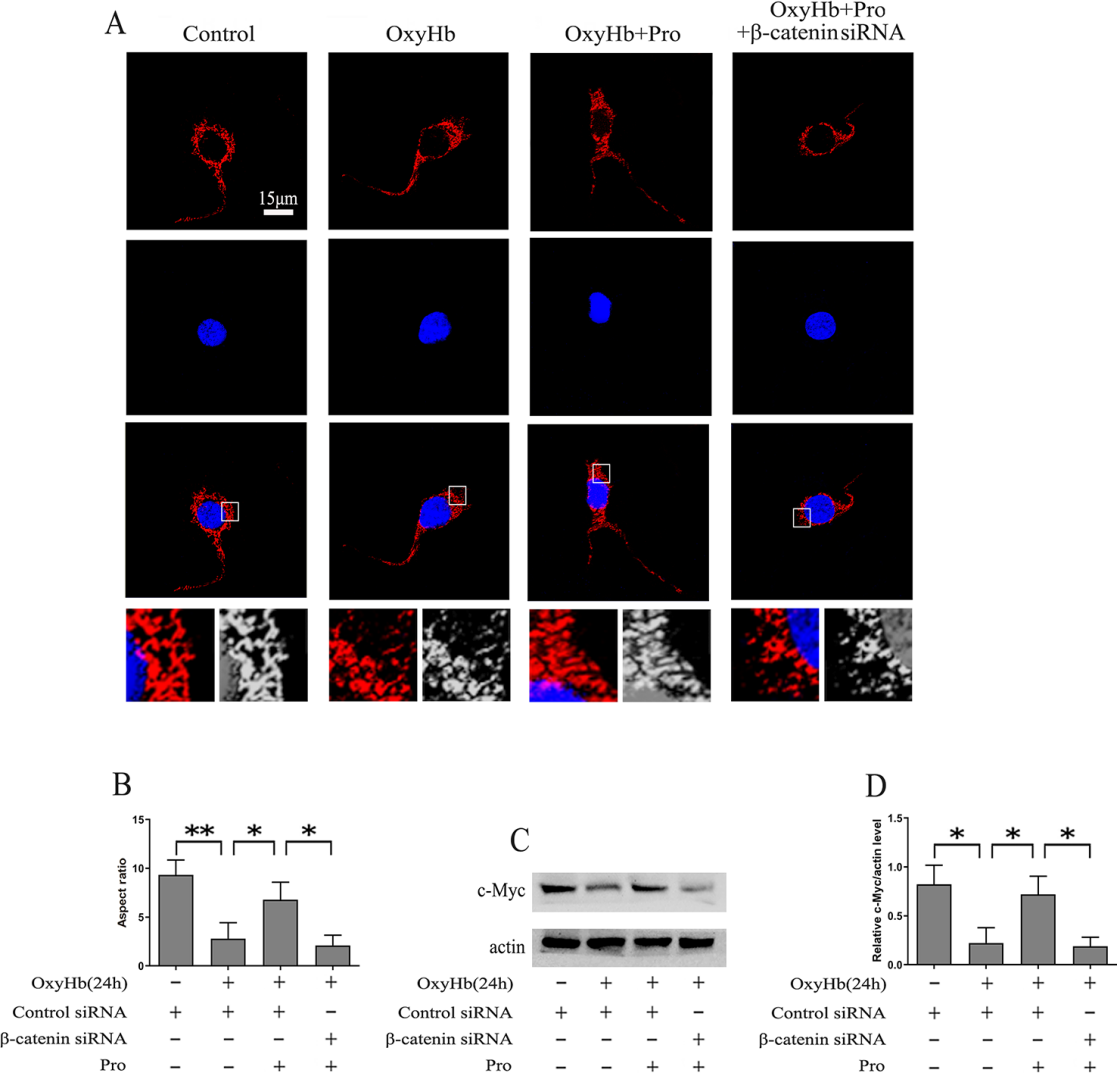
Protective effects of Pro against OxyHb-induced mitochondrial fission in primary cultured neurons. **(A)** MitoTracker was used to stain primary cultured neurons treated as described in **Figure 2H** (scale bar, 15 μm). **(B)** The ratio between the major and minor axes of an ellipse equivalent to the mitochondrial shape is the aspect ratio of mitochondria. A total of 10 fields of view in a coverslip were recorded, and the experiments were performed 3 times (mean ± SD; n = 3; *p < 0.05; **p < 0.01). **(C)** Western blot analysis of the c-Myc/actin level in primary cultured neurons treated as described in **Figure 2H**. **(D)** Expression of c-Myc (mean ± SD; n = 3; *p < 0.05).

The level of mitochondrial fission was analyzed using MitoTracker, and the results suggested that OxyHb induced significant mitochondrial fission. After pretreatment with Pro before adding OxyHb, the amount of mitochondrial fission was partially alleviated. However, silencing β-catenin abrogated the protective effect of Pro against the mitochondrial fission phenotype induced by OxyHb (**Figures 4A, B**). c-Myc may modulate the fission and fusion of mitochondria. The Western blots indicated that Pro could reverse the inactivation of c-Myc induced by OxyHb and that knockdown of β-catenin largely abrogated this positive effect of Pro in primary culture neurons (**Figures 4C, D**).

## Discussion

EBI induced by a decrease of cerebral blood flow is an important pathological feature and primary cause of poor prognosis in tSAH (Chen et al., 2018). A common result of EBI is neuronal apoptosis, which induces disruption of the blood-brain barrier, oxidative stress, brain edema, and elevated intracranial pressure (Zhang et al., 2017; Ye et al., 2018). It has been reported that the inactivation of Wnt/β-catenin signaling is associated with neuronal apoptosis (Takadera et al., 2012). In the present study, we found that tSAH significantly induced neuronal apoptosis *in vitro*, as evidenced by the up-regulation of cleaved caspase-8 and down-regulation of the Bcl2/Bax ratio. Further, we found a marked inactivation of Wnt/β-catenin signaling in the primary cultured neurons following tSAH, as indicated by the loss of active-β-catenin.

Dunbar et al. have shown that Wnt/β-catenin signaling is activated after opioid treatment in the rat cerebral cortex (Dunbar et al., 2006). Propoxyphene, a weak opioid analgesic drug, is used in the treatment of mild pain (Hayes et al., 2015; Larochelle et al., 2015). Our results indicated that the neuronal apoptosis and down-regulation of active-β-catenin induced by OxyHb exposure could by abrogated by pretreatment with Pro. To confirm the modulatory effect of Pro in Wnt/β-catenin signaling-mediated apoptosis following tSAH, we pretreated primary cultured neurons with Pro before OxyHb exposure, which was used to mimic tSAH *in vitro*, and used β-catenin siRNA to downregulate the β-catenin expression. The results showed that knockdown of β-catenin largely abrogated the protective effect of Pro against OxyHb-induced activation of cleaved caspase-8, down-regulation of the Bcl2/Bax ratio, and increase of TUNEL-positive cells. Furthermore, the study of morphological changes in rat cortical neurons showed that the loss of condensation of the soma, shrinkage of the neuron, and neuronal arborization induced by OxyHb treatment was attenuated by Pro treatment and that silencing β-catenin largely abrogated the positive effect of Pro. Taken together, the results of the present study demonstrate that inactivation of Wnt/β-catenin signaling is involved in tSAH-induced apoptosis and that Pro protects cortex neurons against tSAH-induced apoptosis *via* modulating the accumulation of active-β-catenin.

Mitochondria are important organelles that participate in many important cellular processes, including the production of ROS, energy metabolism, and apoptosis (Wang et al., 2016). It has been reported that mitochondrial dysfunction participated in the pathological process of SAH models (Chou et al., 2017; Wang Z. et al., 2017). A balance between mitochondrial fusion and fission is crucial to mitochondrial morphology and function (Kawano et al., 1995). The expression of c-Myc can restore the mitochondrial volume and induce increased fusion (Graves et al., 2012). Excessive mitochondrial fission has been shown to be involved in the EBI stage following SAH (Wu et al., 2017). In the present study, silencing β-catenin abrogated the protective effect of Pro against the mitochondrial fission phenotype induced by OxyHb. Further, Pro could reverse the inactivation of c-Myc induced by OxyHb, and the knockdown of β-catenin largely abrogated this positive effect of Pro. We thus hypothesize that Pro protects primary cultured neurons from OxyHb-induced mitochondrial fission in an active-β-catenin-c-Myc-dependent manner.

We conclude that, together, the data presented in this study demonstrate a protective effect of Pro on the EBI after tSAH and suggest the importance of Wnt/β-catenin signaling in this effect. Pro, a weak opioid analgesic drug, attenuates OxyHb-induced mitochondria-dependent apoptosis in an active-β-catenin-c-Myc-dependent manner. Our study demonstrates a new molecular cascade underlying tSAH-induced cell apoptosis and suggests that promoting the accumulation of active-β-catenin is a potential therapeutic strategy for the treatment of tSAH.

## Data Availability Statement

All datasets generated for this study are included in the article/supplementary material.

## Author Contributions

YL, JW, and LL designed this research. HL, ZL and GG wrote the manuscript. HC participated in the TUNEL staining. ZZ performed primary cell culture. TL performed mitochondrial staining and immunofluorescence. XZ carried out the Western blots.

## Funding

This work was supported by grants from the National Natural Science Foundation of China (81401044, 81230043).

## Conflict of Interest

The authors declare that the research was conducted in the absence of any commercial or financial relationships that could be construed as a potential conflict of interest.

## Abbreviations

SAH, subarachnoid hemorrhage; tSAH, traumatic subarachnoid hemorrhage; Pro, propoxyphene; OxyHb, oxyhemoglobin; EBI, early brain injury; DMEM, Dulbecco’s Modified Eagle’s Medium; FBS, fetal bovine serum; SDS-PAGE, sodium dodecyl sulfate-polyacrylamide gel electrophoresis; NC, immobilon nitrocellulose; TUNEL, terminal deoxynucleotidyl transferase dUTP nick-end labeling.
